# Sodium-Glucose Cotransporter-2 Inhibitors and Cardiovascular Protection Among Patients With Type 2 Diabetes Mellitus: A Systematic Review

**DOI:** 10.1155/2024/9985836

**Published:** 2024-05-11

**Authors:** Richard K. Yankah, Eric K. Anku, Vinay Eligar

**Affiliations:** ^1^Diabetes Specialist Clinic, Cape Coast Teaching Hospital, Cape Coast, Ghana; ^2^Dietherapy and Nutrition Unit, Cape Coast Teaching Hospital, P. O. Box CC 1363, Cape Coast, Ghana; ^3^Department of Diabetes and Endocrinology, Imperial College London Diabetes Centre, Abu Dhabi, UAE

**Keywords:** cardiovascular, sodium-glucose cotransporter-2 inhibitors, type 2 diabetes mellitus

## Abstract

**Background:** Accumulating evidence has demonstrated the positive effects of sodium-glucose cotransporter-2 (SGLT2) inhibitors in managing patients with type 2 diabetes mellitus (T2DM). SGLT2 inhibitors protect patients with T2DM from cardiovascular complications and are generally safe.

**Aim:** The aim of this study is to assess the cardiovascular effects of SGLT2 inhibitors in patients with T2DM.

**Methods:** A systematic review was conducted using published English literature in PubMed and Google Scholar databases.

**Results:** Most of the studies showed significant positive cardiovascular effects of SGLT2 inhibitors in patients with and without established cardiovascular disease (CVD). Empagliflozin reduced the risk of cardiovascular death, hospitalization for heart failure (HHF), cardiovascular death or heart failure, and major adverse cardiovascular events (MACE) such as nonfatal stroke, nonfatal myocardial infarction, and cardiovascular death regardless of the number of cardiovascular risk factors. The effects of empagliflozin on cardiovascular events and mortality in patients with coronary artery bypass graft (CABG) were assessed. Further, the efficacy of empagliflozin in three different phenotypic groups, namely, younger patients with shorter duration of T2DM and highest glomerular filtration rate, women without coronary artery disease, and older adults with advanced coronary artery disease plus several comorbidities, was also assessed. The effects of canagliflozin were evaluated in patients with and without a history of CVD and with different body weights, and in those with and without prior heart failure. Treatment with canagliflozin based on multivariable-predicted cardiovascular risk factors prevented heart failure events more than treatment based on glycated hemoglobin and albuminuria alone. The efficacy of dapagliflozin was evaluated in patients with or at risk of atherosclerotic cardiovascular disease (ASCVD), heart failure status, and left ventricular ejection fraction (LVEF), as well as the elderly population. A reduction in HHF or cardiovascular death and insignificant reduction in MACE were noted. Furthermore, significant reduction in the risk of cardiovascular death and all-cause mortality in patients with heart failure with reduced ejection fraction (HFrEF) was also observed. Sotagliflozin was studied for its cardiovascular outcomes in patients with chronic kidney disease with or without albuminuria and resulted in a reduction in cardiovascular-related deaths and HHF.

**Conclusion:** SGLT2 inhibitors have beneficial cardiovascular effects in patients with T2DM and should be incorporated into their management.

## 1. Introduction

Globally, the prevalence of diabetes mellitus is estimated to be 537 million, and it is projected to increase to 783 million by 2045 [1]. Owing to the increasing prevalence of the disease, it has become a significant public health concern for many nations. Diabetes mellitus accounts for a considerable reduction in life expectancy and quality of life. At least 10% of the total health care expenditure in many countries is towards managing diabetes mellitus and its complications [2].

Over 90% of all patients with diabetes mellitus have type 2 diabetes mellitus (T2DM) [1]. Unfortunately, over 70% of patients with T2DM die of cardiovascular complications. Among age-matched patients, those with T2DM have a threefold increased risk of cardiovascular-related mortality compared with those without diabetes mellitus [3]. Significant factors known to elevate the risk of cardiovascular disease (CVD) in patients with T2DM include chronic systemic hypertension, visceral adiposity, reduced insulin sensitivity, and arterial stiffness [4].

Sodium-glucose cotransporter-2 (SGLT2) inhibitors are new glucose-lowering agents that have been reported to reduce the risk of major adverse cardiovascular events (MACE) and improve renal outcomes. SGLT2 inhibitors prevent glucose reabsorption in the proximal tubules of the kidneys. This results in increased urinary glucose excretion, ultimately reducing blood glucose levels [5]. In a state of hyperglycemia, there is upregulation of SGLT2 and the urinary glucose excretion threshold is increased [6]. The reduction of blood glucose levels by SGLT2 inhibitors depends on the estimated glomerular filtration rate (eGFR). Overall, SGLT2 inhibitors reduce HBA1c level by 15.85–12.57 mmol/mol (0.7%–1.0%) and body weight by 2–4 kg [7].

A combination of natriuresis, diuresis, and body weight reduction by SGLT2 inhibitors partly reduces systolic and diastolic blood pressure (DBP) by 4–5 and 1–2 mmHg, respectively [8].

The accumulation of visceral fat is associated with chronic inflammation and the release of proinflammatory adipokines which contribute to insulin resistance and cardiovascular risk. SGLT2 inhibitors improve the cardiometabolic risk profiles of people with T2DM by reducing fat mass and proinflammatory cytokines, such as leptin and interleukin-6 [9]. Epicardial adipose tissue accumulation and inflammation may result in atherosclerotic CVD, including heart failure with a preserved ejection fraction [9]. SGLT2 inhibitors may disrupt these pathological processes through their effects on adipose tissue.

Arterial stiffness is strongly associated with the development of CVD such as hypertension and heart failure [9]. Furthermore, endothelial dysfunction contributes significantly to the development of coronary artery disease and heart failure [9]. Several studies have demonstrated that treatment with SGLT2 inhibitors reduces aortic stiffness and improves endothelial function [9, 10].

SGLT2 inhibitors are generally safe; however, several adverse effects have been identified. Common adverse effects are genital/urinary tract infections and volume depletion which may lead to dehydration, hypotension, and syncopal attacks [11]. These effects are usually mild and can be easily managed. Other identified adverse effects of SGLT2 inhibitors include diabetic ketoacidosis, acute kidney injury (AKI), bone fracture, lower limb amputation, and Fournier's gangrene [11].

This study highlights the cardiovascular effects of SGLT2 inhibitors in T2DM patients in varied circumstances and adds to the growing body of evidence. It also highlights the common adverse effects of SGLT2 inhibitors.

## 2. Methods

**Figure 1:**
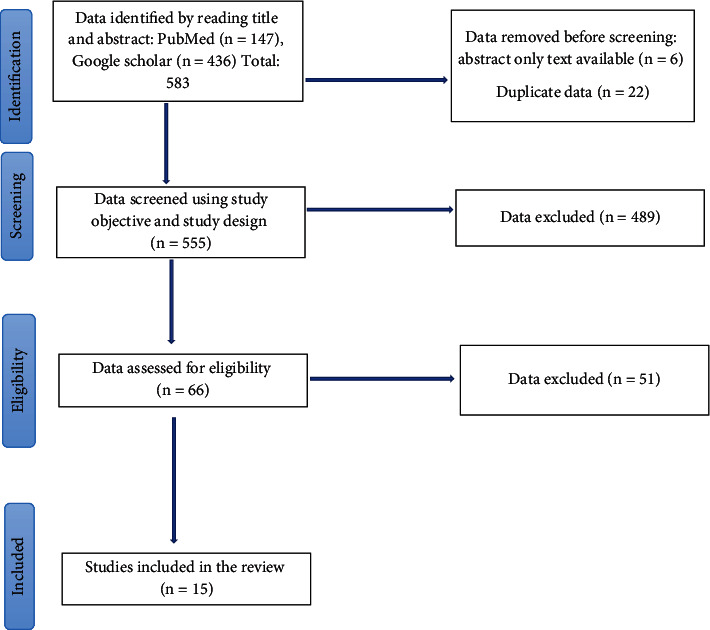
PRISMA search process.

The Preferred Reporting Items for Systematic Reviews and Meta-Analyses (PRISMA) guidelines were used to conduct this systematic review.  Figure 1 illustrates a flow diagram of the search process as per the PRISMA guidelines.

### 2.1. Source of Data and Search Strategy

A systematic search of relevant articles was conducted using PubMed and Google Scholar databases. A literature search was performed using a combination of keywords and medical subject headings (MeSH). The following keywords were systematically combined in the search strategy: T2DM, SGLT2 inhibitors, CVD, MACE, coronary heart disease, myocardial infarction, heart failure, cerebrovascular disease, mortality, and safety.

### 2.2. Inclusion and Exclusion Criteria

The included studies were randomized controlled trials (RCTs) in English published from January 2010 to December 2023 and assessed the cardiovascular effects of SGLT2 inhibitors among patients with T2DM with at least 2000 study participants.

Studies published in languages other than English, those conducted among participants with other types of diabetes mellitus apart from T2DM, and those published before January 2010 were excluded. Studies that were not RCTs were also excluded.

## 3. Results

The initial search yielded 583 articles. Duplicates (*n* = 22) and articles that did not have full texts available (*n* = 6) were removed, leaving 555 articles. This was then screened using the study objective and design to further remove 489 articles, leaving 66 articles. Eligibility criteria were then applied to further remove 51 articles. This study includes 15 articles of RCTs involving 55,501 patients with T2DM treated with SGLT2 inhibitors. The included RCTs studied SGLT2 inhibitors such as empagliflozin, canagliflozin, dapagliflozin, sotagliflozin, and ertugliflozin.

Overall, SGLT2 inhibitors reduced the risk of hospitalization for heart failure (HHF), heart failure, cardiovascular mortality, all-cause mortality, nonfatal myocardial infarction, and nonfatal stroke. SGLT2 inhibitors were found to be generally well tolerated in spite of the predominant adverse effects of genital tract infection and volume depletion.

Table 1 depicts the main characteristics of the included studies.

## 4. Discussion

### 4.1. Cardiovascular Effects of SGLT2 Inhibitors

Overall, SGLT2 inhibitors from various studies have demonstrated beneficial cardiovascular outcomes in T2DM patients. Canagliflozin substantially reduced MACE (nonfatal MI, nonfatal stroke, or cardiovascular death) in patients with different body mass indices. It was also able to reduce MACE in both the primary and the secondary prevention of cardiovascular events. Furthermore, canagliflozin demonstrated consistency in reducing HHF in three studies. Dapagliflozin showed a consistent reduction in HHF or cardiovascular death in three studies, but no significant reduction in MACE was observed with its use. Empagliflozin can reduce the risk of MACE or HHF in patients with varied cardiovascular risk profiles and in those with established CVD. However, secondary prevention in patients who underwent coronary artery bypass graft (CABG) did not reduce the risk of stroke or MI in these patients. In the study by Inzucchi et al. [12], empagliflozin reduced the risk of cardiovascular death, HHF, cardiovascular death or heart failure, and MACE, irrespective of the number of cardiovascular risk factors controlled at baseline. An inherent property of SGLT2 inhibitors other than glycemic control might explain this effect in patients with T2DM. In a short-term study of sotagliflozin, beneficial effects on reducing death from cardiovascular causes and HHF were observed. For a long-term study of this drug, it is important to properly ascertain its cardiovascular effects in patients with type 2 diabetes.

The findings of this study demonstrate that SGLT2 inhibitors significantly reduce the risk of cardiovascular death, HHF, all-cause death, and MACE. These findings are consistent with that of Bhattarai et al. who studied associations of SGLT2 inhibitors and cardiovascular outcomes in patients with T2DM and other CVD risk factors. There was a statistically significant reduction in MACE by empagliflozin and canagliflozin across varied CVD risk factors [13].

In a post hoc analysis of the Empagliflozin Cardiovascular Outcome Event Trial in T2DM patient-removing excess glucose (EMPA-REG) trial, Inzucchi et al. assessed the impact of cardiovascular risk factor control on the beneficial effects of empagliflozin. A total of 7020 adult individuals were randomized to receive empagliflozin or placebo [12]. Adult patients aged 18 years and above with T2DM, HBA1c of 53 to 86 mmol/mol (7% to 10%), body mass index (BMI) of 45 kg/m^2^ or less, eGFR of 30 mL/min/1.73 m^2^, and established atherosclerotic cardiovascular disease (ASCVD) were the eligibility criteria. Risk factors for CVD, such as HBA1c below 58 mmol/mol (7.5%), low-density lipoprotein (LDL) cholesterol below 2.59 mmol/L or use of a statin, systolic blood pressure (SBP) below 140 mmHg and DBP below 90 mmHg, use of aspirin, and nonsmoking status, were assessed at baseline and modified with treatment. Empagliflozin treatment led to a reduced risk of cardiovascular death, HHF, cardiovascular death or heart failure, and 3-point MACE (nonfatal myocardial infarction, nonfatal stroke, or cardiovascular death) compared to placebo, irrespective of the number of cardiovascular risk factors controlled at baseline [12].

This study has some limitations. It is worth noting that other cardiovascular risk factors, including genetic factors, inflammation, and socioeconomic background of the patients, were not included in the trial. In addition, the conclusions of this study may not be generalizable to include all patients with T2DM because the study population used had established ASCVD [12].

In a similar study, Davies et al. in 2017 conducted a post hoc analysis of pooled data from a randomized, double-blind, placebo-controlled trial that assessed canagliflozin in patients with type 2 diabetes (*n* = 2313) and a 6-week study involving patients with T2DM and hypertension (*n* = 169) [14]. The participants received canagliflozin 100 or 300 mg as monotherapy or as add-on therapy to metformin, metformin plus sulfonylurea, or metformin plus pioglitazone. The participants were adult females and males aged 18–80 years. Canagliflozin reduced the mean 24-hour SBP and DBP at Week 6 compared to placebo. The mean reductions from baseline values were −4.5, −6.2, and −1.2 mmHg using canagliflozin 100 mg, canagliflozin 300 mg, and placebo, respectively. The mean reductions in mean 24-hour DBP were −2.2, −3.2, and −0.3 mmHg, respectively. Moreover, canagliflozin reduced the mean arterial pressure and pulse pressure compared with placebo at Week 26. These beneficial effects are likely to translate into reduction in arterial stiffness, arterial resistance, improved blood flow, and reduced cardiac workload. These effects of SGLT2 inhibitors, in addition to reducing HBA1c and body weight, will improve cardiovascular outcomes [14]. This study lacked prespecified statistical testing across various subgroups; hence, the conclusions should be applied with caution. Furthermore, the relatively small number of patients with a history of CVD at baseline places a limitation on the comparisons [14].

Mahaffey et al. in 2018 studied the effects of canagliflozin in individuals with and without prior CVD for primary and secondary prevention of CVD in the Canagliflozin Cardiovascular Assessment Study (CANVAS) [15]. It was observed that canagliflozin in comparison with placebo reduced MACE (nonfatal myocardial infarction, nonfatal stroke, and cardiovascular death) in a homogenous manner among participants in the primary and secondary prevention groups, although the absolute reduction was higher in the secondary prevention group [15]. The limitations of this study include a relatively short follow-up period (approximately 3.5 years), nonscreening of patients assigned to the primary prevention group for subclinical atherosclerotic vascular disease, and reliance on investigator-reported inclusion and exclusion criteria for selecting patients into primary and secondary prevention groups without confirmation. In addition, the study was not statistically powered to highlight categorical differences in treatment outcomes among primary and secondary patient populations [15].

In 2020, Ohkuma et al. also assessed whether the effects of canagliflozin on cardiovascular and renal outcomes, body weight, and safety vary with respect to the baseline BMI in the CANVAS. Participants were categorized according to BMI levels less than 25, 25 to less than 30, and ≥ 30 kg/m^2^ [16]. Canagliflozin was noted to substantially reduce the risk of composite cardiovascular events, such as nonfatal myocardial infarction, nonfatal stroke, or cardiovascular death, compared with placebo. Risk reduction is consistent across BMI levels [16].

A limitation of this study was the relatively small number of patients with a BMI below 25 kg/m^2^, which reduced the statistical power to enable clear conclusions to be drawn with respect to the effects of canagliflozin treatment in patients with lean bodies [16].

Using CANVAS, Rådholm et al. in 2018 sought to determine the efficacy and safety of canagliflozin in individuals with and without baseline heart failure. In this study, canagliflozin substantially reduced HHF and cardiovascular death [17]. Furthermore, this reduction was greater in participants with prior history of heart failure than those without previous history of heart failure. However, statistical significance was not strong enough (*P* interaction = 0.029) [17].

Documentation of heart failure at baseline was limited, as there was a lack of data on baseline biomarkers or echocardiography. This implies that the estimated prevalence of established heart failure lacked perfection; hence, the categorization of patients as having heart failure at baseline may not have been entirely correct. It was also impossible to classify patients as having reduced or preserved heart failure [17].

In 2019, Wiviott et al. evaluated patients with T2DM with elevated risks of ASCVD to determine the effects of dapagliflozin on cardiovascular and renal outcomes in the DECLARE-TIMI 58 study [18]. After a median follow-up of 4.2 years, dapagliflozin was found to be noninferior to placebo with regard to MACE (myocardial infarction, ischemic stroke, or cardiovascular death). Treatment with dapagliflozin did not result in a significant reduction in MACE compared with placebo. Dapagliflozin treatment however resulted in a substantial reduction in the rate of HHF or cardiovascular death (4.9% vs. 5.8%; HR, 0.83; 95% CI, 0.73 to 0.95; *P* = 0.005) [18].

Dapagliflozin was also assessed for its efficacy and safety based on heart failure status and left ventricular ejection fraction (LVEF) in DECLARE-TIMI 58 by Kato et al. in 2019 [19]. Heart failure with reduced ejection fraction (HFrEF) was defined as an ejection fraction of < 45% or moderate/severe left ventricular systolic dysfunction with or without a reported history of heart failure. Treatment with dapagliflozin reduced the risk of cardiovascular death or HHF by 17% (HR, 0.83 (95% CI, 0.73–0.95; *P* = 0.005)). This reduction was observed more in individuals with HFrEF (HR, 0.62 (95% CI, 0.45–0.86)) compared with those without (HR, 0.88 (95% CI, 0.76–1.02; *P* interaction = 0.046)). For participants without HFrEF, the effect estimates of dapagliflozin did not differ between those with heart failure with unknown ejection fraction and those without a history of heart failure. All-cause mortality was also substantially reduced in the participants with HFrEF (HR, 0.59 (95% CI, 0.86–1.1; *P* = 0.01)) compared to those without (HR, 0.97 (95% CI, 0.86–1.10; *P* interaction = 0.016)). The beneficial effects of dapagliflozin in reducing cardiovascular death, HHF, and all-cause mortality in participants with HFrEF occurred early and spanned the entire trial period [19]. The outcomes of this study are consistent with those of the Empagliflozin Outcome Trial in Patients with Chronic Heart Failure and Reduced Ejection Fraction (EMPEROR-Reduced) trial conducted by Parker et al. in 2021 [20] with regard to reduction in HHF or cardiovascular death.

In a study parallel to the EMPEROR-Reduced trial, the EMPEROR-Preserved trial also reported a reduced risk of HHF or cardiovascular death in participants with and without diabetes after a median follow-up period of 26.2 months. A significant reduction in HHF of 29% mainly accounts for this positive outcome [21].

In 2020, Cahn et al. studied the efficacy and safety of dapagliflozin based on age variation [22]. Participants were categorized into those below 65 years, 65 to 74 years, and 75 years and above. Dapagliflozin treatment consistently reduced composite cardiovascular death or HHF across various age groups compared with placebo. However, MACE was not significantly reduced in the dapagliflozin group. Moreover, major hypoglycemic episodes were less frequent in the dapagliflozin group than in the placebo group [22].

One limitation of this study is that patients with creatinine clearance below 60 mL/min/1.73 m^2^ were excluded from the study which may have excluded elderly patients who are likely to have volume depletion, AKI, fractures, etc. Furthermore, cognitive function, frailty, and functional capacity were not assessed at baseline or any time during the study period. Additionally, metabolic profiles were analyzed post hoc and should be considered with caution [22].

The effects of empagliflozin on cardiovascular events and safety in patients with T2DM and self-reported CABG surgery were evaluated by Verma et al. in 2018 [23]. Of the 7020 participants, 25% (1175/4687) who received empagliflozin and 24% (563/2333) who received placebo had a history of CABG surgery in 2018. In this post hoc subanalysis of data from the EMPA-REG OUTCOME trial, empagliflozin reduced cardiovascular death by 48%, all-cause mortality by 43%, HHF by 50%, and incident or worsening nephropathy by 35% compared with placebo. No difference was observed in the risk of myocardial infarction or stroke in participants with or without a history of CABG [23]. The findings should be applied with caution because of the post hoc nature of the analyses. No baseline echocardiography was performed, and no baseline parameters of left ventricular systolic or diastolic function were known. Additionally, the analyses did not adjust for changes in background cardiovascular and glucose-lowering therapies [23].

Another SGLT2 inhibitor, sotagliflozin, has been studied for its cardiovascular effects in patients with T2DM and chronic kidney disease with or without albuminuria for a median period of 15.9 months by Bhat et al. in 2021 [24]. Treatment with sotagliflozin resulted in a reduced risk of composite deaths from cardiovascular causes, HHF, and urgent clinical visits for heart failure (5.6 events per 100 patient-years) compared with placebo (7.5 events per 100 patient-years) (HR, 0.74; 95% CI, 0.63 to 0.88; *P* < 0.001). However, with respect to the rate of death from cardiovascular causes, there was no significant difference between the sotagliflozin and placebo groups [24]. A major limitation of this study is the shorter follow-up time; hence, there is a need to conduct a longer study on this medication to ascertain its long-term cardiovascular effects [24].

In a multicentre study by Cannon et al. in 2020, 8238 adult patients with T2DM and established CVD such as coronary artery disease (75.9%), CVD (22.9%), peripheral artery disease (PAD) (18.7%), and history of heart failure (23.7%) were randomized in a 1 : 1 : 1 ratio to receive once-daily ertugliflozin 5 or 15 mg or placebo and followed up for a mean period of 3.5 years [25]. The mean duration of diabetes mellitus was 13.0 years. Ertugliflozin was shown to be noninferior to placebo with respect to MACE (nonfatal myocardial infarction, nonfatal stroke, or death from cardiovascular causes (HR, 0.97; 95.6% CI 0.85 to 1.11; *P* < 0.001). For deaths from cardiovascular causes or HHF, no significant difference was noted between the ertugliflozin and placebo groups. Ertugliflozin was not superior to the placebo in this study [25]. A limitation of this study was that the secondary outcome of HHF was not statistically tested [25].

Sharma et al. conducted a post hoc study of the EMPA-REG OUTCOME trial in 2021 to determine the effects of empagliflozin treatment in three different phenotypic groups of patients using latent class analysis [26]. Three phenotypic groups comprising patients with type 2 diabetes, various cardiovascular risk profiles, and baseline characteristics were identified. The identified groups were designated as the training group (Group 1) and validation groups (Groups 2 and 3), totalled 6639. Phenotype Group 1 comprised younger patients who had a shorter duration of T2DM and a higher glomerular filtration rate (*n* = 1463, 33.1%); phenotype Group 2 comprised more women with no coronary artery disease (*n* = 1172, 26.5%), and phenotype Group 3 comprised older adult patients with advanced coronary artery disease and more comorbidities (*n* = 1785, 40.4%). Compared with placebo, empagliflozin reduced the risk of cardiovascular death or HHF and cardiovascular death consistently across phenotypic groups [26]. As a limitation, the findings of this study should be applied cautiously as the analyses were a post hoc analysis without a prespecified subgroup analysis plan [26].

Abdul-Ghani et al. in 2016 pointed out the benefit of the EMPA-REG OUTCOME trial which was done in patients with T2DM having elevated CVD risk and established CVD [8]. MACE (nonfatal myocardial infarction, stroke, and cardiovascular death) was reduced by 14%. This beneficial effect was mainly due to a 38% reduction in cardiovascular mortality because the reduction in nonfatal myocardial infarction and stroke was not significant. Furthermore, a 35% reduction in HHF was noted in patients treated with empagliflozin, but no effect was noted with regard to hospitalization for unstable angina. All-cause mortality was reduced by 32% [8]. The reduction in CVD risk is more likely to be due to the hemodynamic effects of empagliflozin, such as reduction in extracellular fluid volume and blood pressure, and less likely due to the reduction in metabolic effects of drugs such as HBA1c, body weight, blood pressure, and elevation of high-density lipoprotein (HDL) cholesterol [8].

In a study by Tye et al. conducted in 2022, canagliflozin was commenced for the treatment of T2DM based on a multivariable risk prediction model, as well as a model based on HBA1c or urine albumin to creatinine ratio (UACR) alone [27]. The results revealed a significant reduction in heart failure and cardiovascular death over a 5-year period when canagliflozin was initiated based on a multivariable risk prediction model compared to initiation based on HBA1c or UACR alone. The first limitation of this study is the small number of patients with the inclusion criteria of HBA1c of 10.5% or lower (54 patients) and albuminuria (1037 patients). Therefore, these findings should be interpreted with caution. Second, the risk prediction models were generated and tested within the trial and were not validated externally. Furthermore, the authors did not include the analysis of all-cause mortality because canagliflozin did not show a statistically significant reduction in the risk of all-cause mortality [27].

Zelniker et al. conducted a secondary analysis of the DECLARE-TIMI 58 trial in 2021 to assess the effect of dapagliflozin on cardiovascular outcomes based on baseline kidney function and albuminuria in patients with T2DM. Patients were grouped according to their baseline eGFR (< 60 mL/min/1.73 m^2^ vs. ≥ 60 mL/min/1.73 m^2^) and baseline UACR (< 30 mg/g against ≥ 30 mg/g) and chronic kidney disease markers (designated 0, 1, or 2 based on treatment with ACEIs or ARBs for patients with T2DM and eGFR ≥ 60 mL/min/1.73 m^2^ or UACR ≥ 30 mg/g or both) [28]. The study revealed a more consistent relative risk reduction in cardiovascular outcomes with the use of dapagliflozin, regardless of baseline eGFR and magnitude of albuminuria [28].

Patients with more chronic kidney disease markers showed a substantially greater absolute risk reduction for composite cardiovascular mortality or HHF. The first limitation of this study is that the results should be applied with caution, as it was an exploratory subgroup study. Second, because most patients enrolled in the DECLARE-TIMI 58 trial had an eGFR of at least 60 mL/min/1.73 m^2^, it may be inappropriate to generalize these results to patients with an eGFR lower than 60 mL/min/1.73 m^2^ [28].

Several mechanisms underlie the beneficial cardiovascular effects of SGLT2 inhibitors. The cardiovascular protection of SGLT2 inhibitors may be provided by a reduction in preload through natriuresis and osmotic diuresis as well as a reduction in the afterload through lowering of the blood pressure and vascular resistance [13]. These mechanisms are believed to reduce ventricular demand and improve cardiovascular function. The reduction in hospitalization secondary to heart failure by SGLT2 inhibitors may be explained by their natriuretic and diuretic effects. SGLT2 inhibitors are also believed to inhibit cardiac fibrosis which plays a key role in the pathogenesis of heart failure [13].

### 4.2. Safety Profile of SGLT2 Inhibitors

SGLT2 inhibitors are generally well tolerated but do have some adverse effects. There are a few adverse effects, some of which may be serious.

Canagliflozin increases the risk of diabetic ketoacidosis, amputation, genital infections, and volume depletion [16]. These effects were consistent across the various BMI categories. However, the risk of urinary tract infection was higher in patients with a baseline BMI of 25 kg/m^2^ to BMI below 30 kg/m^2^ than in those with BMI categories below 25 kg/m^2^ and above 30 kg/m^2^ [16].

In a study by Davies et al. in 2017, adverse effects of canagliflozin were well tolerated and included genital mycotic infections and volume depletion-associated adverse effects such as postural dizziness, hypotension, and orthostatic hypotension. All adverse effects were tolerable with management and did not lead to drug discontinuation [14]. Osmotic diuresis–associated events were substantially lower in patients with a history of heart failure than in those without [17]. Higher rates of diabetic ketoacidosis and genital tract infections were also observed in patients treated with dapagliflozin than in those treated with a placebo [18].

Adverse events noted in a study by Cahn et al. in 2020 included genital infections, volume depletion, acute renal failure (ARF), AKI, and lower limb amputations. The proportion of patients with genital infections was greater in the empagliflozin group than that in the placebo group. Furthermore, the proportion of patients with volume depletion was higher in those with a history of CABG but was similar in the empagliflozin and placebo groups. In addition, the proportion of patients with ARF, including AKI, was lower in the empagliflozin group (5.8%) than in the placebo group (11%). The incidence of lower limb amputations was also similar in both the empagliflozin and placebo groups among patients with and without a history of CABG. Notably, no statistical tests were performed for these observed adverse effects [22].

The significant adverse effects noted with the use of sotagliflozin in comparison with placebo were diarrhea and diabetic ketoacidosis. Sotagliflozin inhibits the SGLT1 and SGLT2 receptors. The inhibitory effect of SGLT1 proteins, which are located in the intestines, results in diarrhea. The differences between sotagliflozin and placebo with regard to other adverse effects such as bone fractures, amputations, urinary tract infections, AKI, and severe hypoglycemia were insignificant [24].

### 4.3. Limitations

The limitations of this study include variations in the characteristics of the study populations, the eligibility criteria used, the follow-up duration of the trials, and varied clinical settings. These limitations might have introduced bias in the results, and as such, interpretation of the results should be performed accordingly. In addition, the study was limited to the English language literature; hence, other relevant studies might have been excluded. Furthermore, the use of only two search engines limited the scope of the search and might have excluded other useful studies.

The review concentrated on the cardiovascular effects of SGLT2 inhibitors, excluding other outcomes, such as renal effects.

## 5. Conclusion

SGLT2 inhibitors are oral glucose-lowering medications used to manage T2DM. They also have modest effects on blood pressure and body weight reduction. Treatment with SGLT2 inhibitors in patients with T2DM has beneficial cardiovascular effects in patients with cardiovascular risk factors and in those with established CVD. However, this beneficial effect is predominant in patients with established CVD. There are some differences in these beneficial cardiovascular effects with respect to the different SGLT2 inhibitors, which may be due to their different molecular structures, affinity to SGLT2 receptors, and/or study designs.

There was a consistent and predominant association between SGLT2 inhibitor use and reduction in HHF among the different patient groups in most of the trials.

This review of RCTs not only adds to previous studies but also presents additional subgroup analyses such as cardiovascular effects of empagliflozin on cardiovascular risk factor control, canagliflozin for primary and secondary cardiovascular risk factor control, effects of canagliflozin on different body mass indices, effects of dapagliflozin on different age categories, and effects of empagliflozin on type 2 diabetes patients who have undergone CABG surgery.

The results of this study support accumulating evidence that SGLT2 inhibitors have beneficial cardiovascular outcomes. SGLT2 inhibitors are effective and safe in the treatment of patients with T2DM.

## Figures and Tables

**Table 1:** Summary of studies and their cardiovascular outcomes.

**Authors**	**Year of publication**	**Drug**	**Number of patients**	**Purpose**	**Result**
Inzucchi et al.	2020	Empagliflozin	7020	Evaluate the cardiovascular benefits across a spectrum of cardiovascular risk factors control	Reduction in risk of cardiovascular death, HHF, cardiovascular death or HHF, and 3-point MACE
Davies et al.	2017	Canagliflozin	2313	Determine the efficacy and safety of canagliflozin in T2DM patients with history of CVD risk factors	Consistent reductions in HBA1c, body weight, and systolic blood pressure in patients with CVD or CVD risk factors
Mahaffey et al.	2018	Canagliflozin	10,142	Determine the effects of canagliflozin in patients with and without prior CVD (primary and secondary prevention)	Reduction in MACE in both primary and secondary prevention groups
Okuma et al.	2020	Canagliflozin	10,142	Assess whether canagliflozin's effects on cardiovascular and renal outcomes, body weight, and safety vary with respect to baseline BMI	Canagliflozin significantly lowered risk of MACE across various BMI categories
Rådholm et al.	2018	Canagliflozin	10,142	Determine the efficacy and safety in patients with and without baseline heart failure	Substantial reduction in HHF or cardiovascular death in patients with history of prior heart failure compared with those without
Wiviott et al.	2019	Dapagliflozin	17,160	Evaluate drug's effects on CV and renal outcomes among patients with or at risk of ASCVD	Insignificant reduction in MACE and significant reduction in HHF or cardiovascular death
Kato et al.	2019	Dapagliflozin	17,160	Efficacy and safety of dapagliflozin based on heart failure status and LVEF level	Significant reduction in the risk of cardiovascular death or HHF or all-cause mortality in patients with heart failure with reduced ejection fraction (HFrEF) than those without
Cahn et al.	2020	Dapagliflozin	17,160	Assess the efficacy and safety of drug in the elderly population in DECLARE-TIMI 58 study	Consistent reduction in cardiovascular death or HHF across the various age groups. Reduction in MACE was not significant
Verma et al.	2018	Empagliflozin	7020	Evaluate the effects of drug on CV events and mortality in patients with T2DM and self-reported CABG	Reduction of cardiovascular death, all-cause mortality, and HHF in study group compared with placebo. No difference in risk of MI or stroke in patients with or without history of CABG
Bhat et al.	2021	Sotagliflozin	10,584	Evaluate CV and renal outcomes of sotagliflozin in patients with T2DM and CKD with or without albuminuria	Reduced risk of composite total deaths from CV causes, HHF, and urgent visits for heart failure. No difference in the rate of death
Cannon et al.	2020	Ertugliflozin	8238	Determine long-term effects of drug on CV and renal outcomes	Ertugliflozin was noninferior to placebo with respect to MACE
Sharma et al.	2021	Empagliflozin	6639	Determine the effect of empagliflozin treatment in three different phenotypic groups of patients with T2DM and different cardiovascular risk profiles	Treatment with empagliflozin consistently reduced the risk of cardiovascular death, HHF, and cardiovascular death across the three groups
Abdul-Ghani et al.	2016	Empagliflozin	7020	Explored possible mechanisms underlying the beneficial effects of empagliflozin and implications for their use	The reduction in the risk of cardiovascular events is more likely due to hemodynamic effects of empagliflozin (reduced extracellular volume and blood pressure) and less likely due to metabolic effects (reduction in HbA1c and body weight and increase in HDL cholesterol)
Tye et al.	2022	Canagliflozin	3713	Examine the effect of canagliflozin treatment initiation in the prevention of heart and kidney failure events guided by multivariable-predicted risk factors compared with treatment guided by HBA1c or albuminuria alone	Treatment based on multivariable risk prediction model results in better outcomes in terms of prevention of heart failure events than a treatment based on HBA1c or albuminuria alone
Zelniker et al.	2021	Dapagliflozin	17,160	Determine the effect of cardiovascular outcomes according to baseline kidney function and albuminuria	There was a consistent relative risk reduction in cardiovascular outcomes regardless of baseline eGFR and level of albuminuria

## References

[1] International Diabetes Federation IDF Diabetes Atlas. 10th ed. 2021.1-141. https://diabetesatlas.org/idfawp/resource-files/2021/07/IDF_Atlas_10th_Edition_2021.pdf.

[2] O’Connor P. J., Crain A. L., Rush W. A., Hanson A. M., Fischer L. R., Kluznik J. C. (2009). Does diabetes double the risk of depression?. *Annals of Family Medicine*.

[3] US Department of Health and Human Services (2020). National Diabetes Statistics Report, 2020. *National Diabetes Statistics Report*.

[4] Inzucchi S. E., Bergenstal R. M., Buse J. B. (2015). Management of hyperglycemia in type 2 diabetes, 2015: a patient-centered approach: update to a position statement of the american diabetes association and the european association for the study of diabetes. *Diabetes Care*.

[5] Marsenic O. (2009). Glucose control by the kidney: an emerging target in diabetes. *American Journal of Kidney Diseases*.

[6] Zhao Y., Gao P., Sun F. (2016). Sodium intake regulates glucose homeostasis through the PPAR*δ*/adiponectin-mediated SGLT2 pathway. *Cell Metabolism*.

[7] van Bommel E. J. M., Muskiet M. H. A., Tonneijck L., Kramer M. H. H., Nieuwdorp M., van Raalte D. H. (2017). SGLT2 inhibition in the diabetic kidney-from mechanisms to clinical outcome. *Clinical Journal of the American Society of Nephrology*.

[8] Abdul-Ghani M., Del Prato S., Chilton R., De Fronzo R. A. (2016). SGLT2 inhibitors and cardiovascular risk: lessons learned from the EMPA-REG outcome study. *Diabetes Care*.

[9] Palmiero G., Cesaro A., Vetrano E. (2021). Impact of SGLT2 inhibitors on heart failure: From pathophysiology to clinical effects. *International Journal of Molecular Sciences*.

[10] Jhalani N. B. (2022). Clinical considerations for use of SGLT2 inhibitor therapy in patients with heart failure and reduced ejection fraction: a review. *Advances in Therapy*.

[11] McGill J. B., Subramanian S. (2019). Safety of sodium-glucose co-transporter 2 inhibitors. *The American Journal of Medicine*.

[12] Inzucchi S. E., Khunti K., Fitchett D. H. (2020). Cardiovascular benefit of empagliflozin across the spectrum of cardiovascular risk factor control in the EMPA-REG OUTCOME trial. *The Journal of Clinical Endocrinology and Metabolism*.

[13] Bhattarai M., Salih M., Regmi M. (2022). Association of sodium-glucose cotransporter 2 inhibitors with cardiovascular outcomes in patients with type 2 diabetes and other risk factors for cardiovascular disease. *JAMA Network Open*.

[14] Davies M. J., Merton K., Vijapurkar U., Yee J., Qiu R. (2017). Efficacy and safety of canagliflozin in patients with type 2 diabetes based on history of cardiovascular disease or cardiovascular risk factors: a post hoc analysis of pooled data. *Cardiovascular Diabetology*.

[15] Mahaffey K. W., Neal B., Perkovic V. (2018). Canagliflozin for primary and secondary prevention of cardiovascular events. *Circulation*.

[16] Ohkuma T., Van Gaal L., Shaw W., Mahaffey K. W., de Zeeuw D., Matthews D. R. (2020). Clinical outcomes with canagliflozin according to baseline body mass index: results from post hoc analyses of the CANVAS program. *Diabetes, Obesity and Metabolism*.

[17] Rådholm K., Figtree G., Perkovic V. (2018). Canagliflozin and heart failure in type 2 diabetes mellitus: results from the CANVAS program. *Circulation*.

[18] Wiviott S. D., Raz I., Bonaca M. P. (2019). Dapagliflozin and cardiovascular outcomes in type 2 diabetes. *The New England Journal of Medicine*.

[19] Kato E. T., Silverman M. G., Mosenzon O. (2019). Effect of dapagliflozin on heart failure and mortality in Type 2 diabetes mellitus. *Circulation*.

[20] Parker M., Anker S. D., Buttler J. (2021). Empagliflozin in patients with heart failure, reduced ejection fraction, and volume Overload. *Journal of the American College of Cardiology*.

[21] Anker S. D., Butler J., Filippatos G. (2021). Empagliflozin in heart failure with a preserved ejection fraction. *New England Journal of Medicine*.

[22] Cahn A., Mosenzon O., Wiviott S. D. (2020). Efficacy and safety of dapagliflozin in the elderly: analysis from the DECLARE-TIMI 58 study. *Diabetes Care*.

[23] Verma S., Mazer C. D., Fitchett D. (2018). Empagliflozin reduces cardiovascular events, mortality and renal events in participants with type 2 diabetes after coronary artery bypass graft surgery: subanalysis of the EMPA-REG OUTCOME® randomised trial. *Diabetologia*.

[24] Bhatt D. L., Szarek M., Pitt B. (2021). Sotagliflozin in patients with diabetes and chronic kidney disease. *The New England Journal of Medicine*.

[25] Cannon C. P., Pratley R., Dagogo-Jack S. (2020). Cardiovascular outcomes with ertugliflozin in type 2 diabetes. *The New England Journal of Medicine*.

[26] Sharma A., Ofstad A. P., Ahmad T. (2021). Patient phenotypes and SGLT-2 inhibition in type 2 diabetes: insights from the EMPA-REG OUTCOME trial. *JACC: Heart Failure*.

[27] Tye S. C., Jongs N., Coca S. G. (2022). Initiation of the SGLT2 inhibitor canagliflozin to prevent kidney and heart failure outcomes guided by HbA1c, albuminuria, and predicted risk of kidney failure. *Cardiovascular Diabetology*.

[28] Zelniker T. A., Raz I., Mosenzon O. (2021). Effect of dapagliflozin on cardiovascular outcomes according to baseline kidney function and albuminuria status in patients With type 2 diabetes: a prespecified secondary analysis of a randomized clinical trial. *JAMA Cardiology*.

